# Supplementary material for: Refining pairwise sequence alignments of membrane proteins by the incorporation of anchors
Source: PLoS One. 2021 Apr 30;16(4):e0239881. doi: 10.1371/journal.pone.0239881 (PMC8087094; doi:10.1371/journal.pone.0239881)
Supplement: Attachment — Submitted filename: Response to reviewers.pdf [file pone.0239881.s003.pdf]

We thank the reviewers and the editor for their time and valuable feedback. We respond to the reviewer's comments in blue below. The manuscript text has been extensively modified; relevant changes are marked in red, and 3 new figures have been added (Fig. 4B, S1 and S2).

Reviewer #1

The authors describe a new feature that they implemented in the AlignMe software, the anchoring of residues, to improve pairwise alignments of membrane proteins of remote homology. The manuscript is well written, clearly presented and interesting. The functionality in itself has merit. I have the following comments regarding the manuscript:

1. My understanding is that anchoring isn't new, and that the novelty described in this paper is the implementation of such an algorithm in AlignMe. The introduction is extensive and interesting for a general audience, but nearly gives the impression that anchoring is the novelty described in this manuscript. So do the results, which are interesting to see, but don't show any novel insight on the protein sequences taken as input (the performance of AlignMe being the novelty). The authors should clarify the scope of the study.

The reviewer is correct that the novelty is primarily the implementation of anchors in AlignMe, which makes our software the only membrane protein-specific tool for which this feature is available. The results illustrate how it can be applied, which we feel will be important for the typical biochemist who is likely unaware that this feature exists elsewhere and also what the implications might be for their study. We have rewritten the Abstract, Introduction and Discussion to clarify the scope of the study. We anticipate that this will address some of the comments from the other reviewers as well (see below).

2. The introduction should describe ways anchors can be determined. My understanding is that all the anchors used in this manuscript are derived from structural data. Is this true in general, or are there other ways one can do it?

The anchors in this manuscript are, and could be, derived from different sources. In the case of BetP aligned to LeuT, for example, the restraints were selected precisely because they were supported by biochemical data determined independently of structural data. In our study, the structural data was used to provide a global score of the resultant alignments. We have revised the text in the Introduction (p4), Methods (p7), and Results (e.g., p8) to clarify this point.

3. In terms of reproducibility, I don't see how anchors fundamentally differ from manual adjustments. In the end, one leverages external evidence not visible from the sequence itself to refine the alignment. Can the authors clarify exactly how they think anchors improve reproducibility over manual adjustments?

The reviewer raises an important point. When adjusting an alignment to match two positions, sections of sequence downstream and upstream of that position will also necessarily change. The matching of these more remote regions, as well as the definition of the boundaries of the adjusted region, will need to be determined *ad hoc*. Consider the extreme case of a single

anchored position toward the end of the alignment of BetP and LeuT; how specifically should one align the preceding nine helices, given that their similarity is almost negligible? When using AlignMe with anchors, the alignment algorithm makes those choices instead of the user, thereby improving reproducibility. We have revised the Abstract and Introduction to clarify this point.

4. The authors claim that "new, anchored alignments, [...] could potentially contain mechanistic insights [...]". However if my understanding that anchors are mainly determined from structural evidence is correct, I don't see what additional insight the alignment could provide, that wouldn't have been visible from the experimental evidence itself.

As mentioned above, we expect that anchor positions will be derived from information other than global structural alignments (which are only necessary here as a reference for which the answer is known). Anchors derived from non-structural data, or, from structural data describing a fragment of the protein sequence, can therefore be used to create alignments that may provide novel insights in other regions of the alignment. Using the example of BetP-LeuT alignment, a single anchor would allow matching of the other eleven transmembrane helices, which in turn could be essential for identifying other binding site residues or functional features.

5. Clearly anchoring provides significant improvements over naive pairwise alignment methods. However we live in the age of deep sequencing. Tools such as HH-suite proved very effective at identifying even remote homology in a fully automated manner. They can accurately identify conserved residues even in very remote homology cases without the need for manual intervention. It would be relevant to compare the results of anchored pairwise alignments with more challenging methods like this, for instance using pairwise HHMs with sequence profiles built on deep MSAs. Does anchoring still provide more accurate alignments in this scenario? In addition AlignMe has a profile-profile mode. Why was this not used as a comparison point? It should be straightforward to obtain deep MSAs for all the sequences used in this paper. This should be doable for both the leuT and SERCA examples.

It is certainly welcome that the availability of deep sequencing enables more accurate detection of remote homology or of conserved residues. Nevertheless, these approaches do not, in our opinion, negate the need for additional tools for accurately and reproducibly modifying sequence alignments based on external information, as discussed above. In the context of another project in our lab, the EncoMPASS database (encompass.ninds.nih.gov), for example, we need to extend pairwise sequence alignments obtained from symmetry detection algorithms beyond the segments that have been aligned. We therefore regard anchoring as a complementary strategy and as such, a comparison with deep-MSA-based methodologies, or the use of deep sequencing in the profile-profile mode of AlignMe, are beyond the scope of the current manuscript. We hope our revisions to the text make that clear.

6. In addition, the authors may want to describe how to use this feature in practice in AlignMe. As one needs to define custom alignment parameters to add anchors, it would be useful to

provide some guidance on how to set the various parameters. This might also help clarify the scope of this article.

Thank you for this suggestion. In fact, the user only needs to provide a single parameter, beyond the residue selection, and that is the “weight”, which is the contribution of the anchor to the Needleman-Wunsch scoring matrix. To provide some insight into the impact of weights of different values, we now compared alignments obtained with strict enforcement (weight = 1000), with those obtained with lower weights (Fig. 4B), showing that, at least for alignments of BetP and LeuT, weights ~1 would be sufficient to achieve the same alignment (Results, p11). Thus, the extremely high weights (1000) used in the rest of the manuscript serve to strictly enforce matching of the two aligned residues. We have clarified these points in the revised manuscript (e.g., Methods p7), and also explain how we recommend the weights be used (Results p11).

7. The authors write "The above examples are limited by lack of direct structural data", I believe referring to the SERCA pump. However there is a wealth of structural data described in Primeau 2018. Can the authors clarify this sentence?

Thank you for pointing out this oversight. We have revised this section of the Results extensively, and removed this sentence.

8. The authors write "First, optimal forms of input [...]". I don't understand this sentence. What does "Form of input" mean? How does it relate to the manuscript? This reads like a method but I don't see it actually addressed in the methods/results section. How is optimal defined, and what is optimized?

We apologize for the confusion. We were explaining how AlignMe itself was developed, and referred to the various inputs that AlignMe was designed to utilize, the selection of which was optimized to produce accurate alignments. We have rephrased this section of the Introduction.

9. The software doesn't have an open source license, which is against the PLOS ONE guidelines. As the source code is already available on GitHub this should be easy to address.

Thank you, we added a LICENSE to GitHub: github.com/Lucy-Forrest-Lab/AlignMe

Reviewer #2

Existing tools fail to properly align transmembrane proteins due to the large numbers of motif repeats. The authors propose a new approach for their AlignMe tool to improve the alignments of transmembrane proteins. It consists of specifying a pair of residues in the source and the target that must be aligned together, thus constraining the overall alignment. This can cause large offsets, from a default alignment, while still making the final alignment valid. This method is very interesting but requires precise knowledge of the secondary structure of the aligned sequences to define the anchors.

We thank the reviewer for their positive comments, but we were remiss in giving the impression that the secondary structure of the sequences must be known for defining anchors. In fact, the anchored pair of residues can derive from any region of the protein. The requirement is only that they have functional similarity (and thus, presumably an evolutionary relationship). We modified several areas of the manuscript and figure legends (e.g., no longer naming the anchors by transmembrane segment) in an attempt to dispel that impression.

Major revisions:

1. Figure 4 has an axis that misrepresents the distinctions between results with TM1, TM8 or both anchors. Showing that the 20-250 residue shift is present over the entire alignment without the anchors is not very useful. In addition, using colored points is less precise than straight lines.

Unfortunately, we fail to understand what is misleading about the current representation, since it is correct that the entire alignment *is* shifted by 200-250 residues. This format also illustrates that anchors applied in very disparate parts of the protein can have similar effects along the length of the alignment. From the reviewer's comment we are not clear what alternative representation they would prefer. In this case we found that straight lines actually made comparing the effects of different anchors difficult. However, we made the orange circles smaller, for clarity.

Minor revisions:

2. Specify in the abstract and in the intro that the AlignMe tool is available online. Specify in the intro that your team previously developed AlignMe and proposes a new criterion to improve this tool. It is not clear right now.

Thank you for the suggestion. We have added this information in the Abstract and the end of the Methods.

3. Why use the TM1 and TM8 anchors, rather than the others ?

Only two individual anchors are used, each of which matches a single column in TM1 or TM8 of the LeuT fold in both proteins. We chose these two amino acid positions because, as mentioned in the manuscript, they are known to bind Na^+ in both proteins (and in the larger protein superfamily). We feel our choice of naming of the anchors may have been confusing (see also Reviewer 1, #2) and so have modified the names throughout the revised manuscript. We also revised the descriptions of how the anchors were selected in the Results (e.g. p11).

4. Figure 3 lacks text annotations to specify the positions of the anchors and the meaning of the red lines of the anchors.

Thank you, we have added this information to Figure 3.

5. Figure 5 does not show the contribution of each anchor separately. The legend is not clear, it is not necessary to list all the names of the anchors when the count is sufficient. An addition in the figure or an additional table specifying the positions of the anchors and therefore of the shifts that can be corrected would be useful.

We have changed the representation in Figure 5 now to emphasize the need for coverage in the most challenging cases where absolute accuracy is required. The Results have been changed accordingly. We also provided a Supplementary figure of the reference alignment illustrating the positions of the applied restraints (S2 Fig).

6. For all the figures, their markup in the text is brutal and does not allow a correct understanding of their usefulness without referring to the legends of the figures.

We apologize if information was lost in our effort to be concise. We have expanded on the mentions to the figures in the corresponding revised Results sections.

7. Discussion: ‘This is because global alignment methods favor …’ is redundant

Thank you. We have deleted this sentence.

8. This approach could be used to align other types of proteins made up of repeats, such as propellers. Are you planning to make AlignMe available in this type of case?

Although the various modes of AlignMe usage were developed specifically for alignment of membrane proteins, the underlying algorithm is suitable for any type of protein, including propellers, as mentioned in the revised Discussion. Therefore, the anchors feature is available, should the user choose to apply it.

9. For the website: Add a more interactive interface to define the anchors with the possibility of launching a first analysis of the secondary structures to then select the appropriate anchors. Their placement in all the advanced criteria does not make them accessible.

Thank you for the suggestion. We have modified the website so that the Anchors are more prominent and accessible on the main alignment page, and no longer buried in the “detailed alignment parameter” section. See: http://www.bioinfo.mpg.de/AlignMe/AlignMePW.html

We note, however, that analyzing the secondary structures first would be a misleading feature to provide, since using secondary structure is not the only way to identify anchors, as mentioned above. We feel that the confusion may have arisen from our description of the anchors in some of the test cases and have revised the manuscript to prevent this confusion.

Reviewer #3
Overall the manuscript is well written and makes an interesting potential contribution to PLoS One. The work presented is straightforward and somewhat simplistic. The authors could have tested their method and presented their results for additional test cases of increasing difficulty to make their conclusions more compelling. In their results, they only show two examples that work well with their algorithm...

Thank you for the positive comments. However, we would like to point out that there are three already examples provided in the manuscript, all of which are challenging, albeit in different ways. We have expanded the description of the test cases in a number of places (e.g. Results, p11, legends to Figs 3, 4) to clarify the ways in which the anchors may be applied.

1. ... and they do not seem to address that increasing constraints may lead to user bias. Can an incorrect anchor lead to a seemingly correct alignment?

The reviewer's point about increasing numbers of constraints causing user bias is well taken, as poor constraints will necessarily lead to poor alignments. Nevertheless, our goal is to provide the ability to restrain the alignments; we leave it to the user to compare the effects of these restraints and to use caution in their application. We added some discussion of this issue (Results, p11; Discussion p13), encouraging the user to favor anchors at pairs of residues for which the evidence is clearest, and/or to compare the predictions obtained from multiple alternative combinations of anchors, as is now done for the first case study (see Fig. 2 and Fig. S1). We also added an analysis of the effect of the strength of the anchor (Fig. 4B).

2. Additionally, is this algorithm compatible with beta-barrel membrane proteins, which may be worth mentioning or investigating.

As mentioned in response to Reviewer 2 (#8), and in the revised manuscript, the parameters for the most accurate AlignMe modes were optimized for alpha-helical membrane proteins. However, we also expanded the Discussion to mention how AlignMe could be used for beta-barrel membrane proteins or water-soluble proteins, if the user should so choose (p13).

3. Page 6: Authors do make a point for the usefulness of anchoring residues in the alignment. But this is not new and has been used in manual approaches to sequence alignment. What are the criteria for particular residues to be selected for anchoring during alignment? The author's use well-known examples, but potential anchor residues could be more ambiguous (residues that impact function, but the substrate binding residues are unknown). The impact of the weight used for anchor residues would also be informative.

These are important points, raised also by the other Reviewers. Please see responses above regarding the selection of anchor positions (Reviewer 1, #2 and Reviewer 2, #3) and the impact of the weights (Reviewer 1, #6).

4. Figure 2B & C: A better description of the y-axes would help non-expert readers.

Thank you. We expanded on the descriptions of all the figures in the legends and the text.

5. Overall, the figures were poor quality (blurry/pixelated).

We apologize and hope that this did not affect the ability of the reviewer to understand the figures; we are uncertain how this came about, as all the uploaded figures were high-resolution TIFFs. We will certainly ensure that this doesn't happen in the final version.

6. The alignment in Figure 2A is speculative and an alternative alignment has been presented (Anderson et al., Sci Signal 2017). It would have been informative to test both sets of anchoring residues and possibly discern criteria for "correct" versus "incorrect" alignments. The alignment of peptides presents unique challenges.

We fully agree that the alignment of peptides presents unique challenges, and indeed, this was our motivation. We recomputed our alignments using anchors provided by the Anderson et al 2017 alignment, which differ in a few sequence pairs from those in Primeau et al 2018, as used in our earlier version. The anchors are able to reproduce that alignment as well, and so we include those data as supplementary/alternate options (S1 Fig).

We also considered whether these studies could provide criteria for correctness, for example, whether the shift error is more dramatic when comparing against one reference alignment or another. However, ultimately, the inability of the underlying alignment method to obtain the correct alignment *a priori* arises from a complex set of factors and thus the extent of the discrepancy may not be meaningful. Thus, the two sets of alignments should be considered distinct predictions that can be tested experimentally. The value of AlignMe anchoring in this case is to remove arbitrary decisions about the non-anchored positions in the remainder of the alignment.

7. The cases presented in the results appear to be benchmarking cases. What is lacking is one or more unknown cases where sequence and evolutionary relationships are less clear, but functional data (important residues) are available.

The scope of this manuscript, now further clarified according to the suggestions of Reviewer 1 (#1), is to illustrate how this method can be applied and the types of studies where it may be of utility. These examples do, in fact, include those in which the sequence relationships were, at some stage, unclear. However, it is required that we have a known structure alignment for scoring the alignments. We leave it to the readership to find their interesting test cases for which the correct alignment is currently unknown.

8. Clearly, the use of anchors is interesting, but there is no attempt to characterize the quality of the anchor and the impact on AlignMe. For proteins where there is no structural information and mutagenesis data are available, what is the impact of using "fuzzy" anchors.

For that matter, LeuT and BetP could have been compared using “fuzzy” anchors and lower weights to better balance the imposed anchor with the scoring path through the matrix.

Designing the optimal procedure for identifying fuzzy constraints is certainly of interest. However, the impact of an anchor with a non-saturating weight will depend on many factors relating to the input information, including (1) the similarity between the sequences, at every position in their alignment, and (2) the types of descriptors used in that alignment mode, e.g. secondary structure profiles, PSSMs, or transmembrane predictions. Thus, a systematic accounting of fuzzy anchors is a non-trivial goal and is beyond the scope of the current project.

Nevertheless, to clarify the choice of weight values, inspired by the reviewers’ comments (see also Reviewer 1, #6), we now include an analysis of the magnitude of weights for the alignment between BetP and LeuT (Figure 4B). This new analysis illustrates two things: (1) that weights of >10 are likely to be exactly imposed in this case, and (2) even at low weights of ~1, there is a tipping point beyond which the anchors essentially have the same effect across the alignment as a weight of 1000. We therefore encourage the user to apply constraints with saturating weights and only then to compare the effects of weakening those constraints.
